# The Japanese Catheter Ablation Registry (J‐AB): Annual Report in 2024

**DOI:** 10.1002/joa3.70429

**Published:** 2026-07-20

**Authors:** Kengo Kusano, Koichi Inoue, Koshiro Kanaoka, Koji Miyamoto, Yasuo Okumura, Yu‐ki Iwasaki, Kazuhiro Satomi, Seiji Takatsuki, Kohki Nakamura, Seigo Yamashita, Masaharu Masuda, Yoshitaka Iwanaga, Shoko Chishaki‐Kawabata, Teiichi Yamane, Wataru Shimizu, Hiroshi Tada, J‐AB registry investigators

**Affiliations:** ^1^ Department of Cardiovascular Medicine National Cerebral and Cardiovascular Center Suita Japan; ^2^ Cardiovascular Division National Hospital Organization Osaka National Hospital Osaka Japan; ^3^ Department of Medical and Health Information Management National Cerebral and Cardiovascular Center Suita Japan; ^4^ Division of Cardiology, Department of Medicine Nihon University School of Medicine Tokyo Japan; ^5^ Department of Cardiovascular Medicine Nippon Medical School Tokyo Japan; ^6^ Department of Cardiology Tokyo Medical School Tokyo Japan; ^7^ Department of Cardiology Keio University School of Medicine Tokyo Japan; ^8^ Division of Cardiology Gunma Prefectural Cardiovascular Center Maebashi Japan; ^9^ Division of Cardiology, Department of Internal Medicine The Jikei University Katsushika Medical Center Tokyo Japan; ^10^ Cardiovascular Center Kansai Rosai Hospital Amagasaki Japan; ^11^ Department of Cardiology Sakurabashi‐Watanabe Hospital Osaka Japan; ^12^ Division of Cardiology Department of Internal Medicine The Jikei University School of Medicine Tokyo Japan; ^13^ Department of Cardiovascular Medicine New Tokyo Hospital Matsudo Japan; ^14^ Department of Advanced Cardiovascular Therapeutics, Faculty of Medical Sciences University of Fukui Fukui Japan

**Keywords:** catheter ablation, complication, J‐AB, nationwide registry

## Abstract

The Japanese Catheter Ablation (J‐AB) registry, started in August 2017, is a voluntary, nationwide, multicenter, prospective, observational registry performed by the Japanese Heart Rhythm Society (JHRS) in collaboration with the National Cerebral and Cardiovascular Center. From January 2022, the data registration system was changed from Research Electronic Data Capture (REDCap) system to Fountayn system. The purpose of this registry is to collect the details of target arrhythmias, the ablation procedures, including the types of target arrhythmias, outcomes, and acute complications in the real‐world settings. During the year 2024, we have collected a total of 112 151 procedures (mean age of 67.5 years and 65.2% male) from 589 participant hospitals. Detailed data were shown in Figures and Tables.

## Graphical abstract

Analysis of 112 151 catheter ablation procedures from the 2024 Japanese Catheter Ablation (J‐AB) Registry demonstrated consistently low acute complication rates, with a slight reduction from 2023, confirming the safety of contemporary catheter ablation practice in Japan.

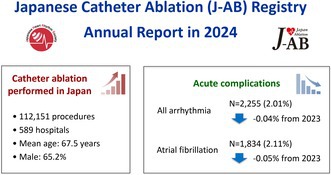

Catheter ablation has become an established therapy for the management of various cardiac arrhythmias, and the procedure number has been dramatically increasing. However, little is known about the details of target arrhythmias, the ablation procedures, including the types of target arrhythmias, outcomes, and acute complications in the real‐world settings.

There are several preceding registries of catheter ablation, but the majority of them collected data from selected centers and/or a selected arrhythmia and/or specified months to reveal the current status of ablations. Accordingly, we conducted a nationwide, multicenter, prospective, observational registry in Japan, named the Japanese Catheter Ablation (J‐AB) registry, aiming to register all catheter ablation cases in Japan. This registry has been performed by the the Japanese Heart Rhythm Society (JHRS) in collaboration with the National Cerebral and Cardiovascular Center using initially Research Electronic Data Capture (REDCap) system [1]. From January 2022, the data registration system was changed from REDCap to Fountayn system, renamed J‐AB 2022, and the research protocol was approved by the central ethics review board of the JHRS (no. 2021001, approved on Dec 16, 2021), and participation is permitted with the approval of the director of each data‐providing institution. All participants were provided informed consent either by a written paper or by an opt out fashion and could withdraw their consent at any time. This study was also registered in the UMIN Clinical Trial Registry (UMIN 000028288) and ClinicalTrials.gov (NCT03729232). This J‐AB registry started in August 2017, since then the number of participating hospitals has increased to over 500 at the end of 2022. Annual data from the year of 2018 to 2023 have already been reported [2, 3, 4, 5, 6, 7] and now we report here the annual report of the results during the year of 2024. Figure 1 showed that the cumulative procedures during the year of 2024. Figure 2 showed that the number and rate of the target arrhythmias. AF ablation was the leading procedure (77.4% of all ablation procedures) in 2024, and the percentage of patients over 75 years of age was 35.9% in 2024. Patient characteristics, acute outcomes, and acute complications of all and AF procedures are shown in Tables 1, 2, 3, respectively.

**FIGURE 1:**
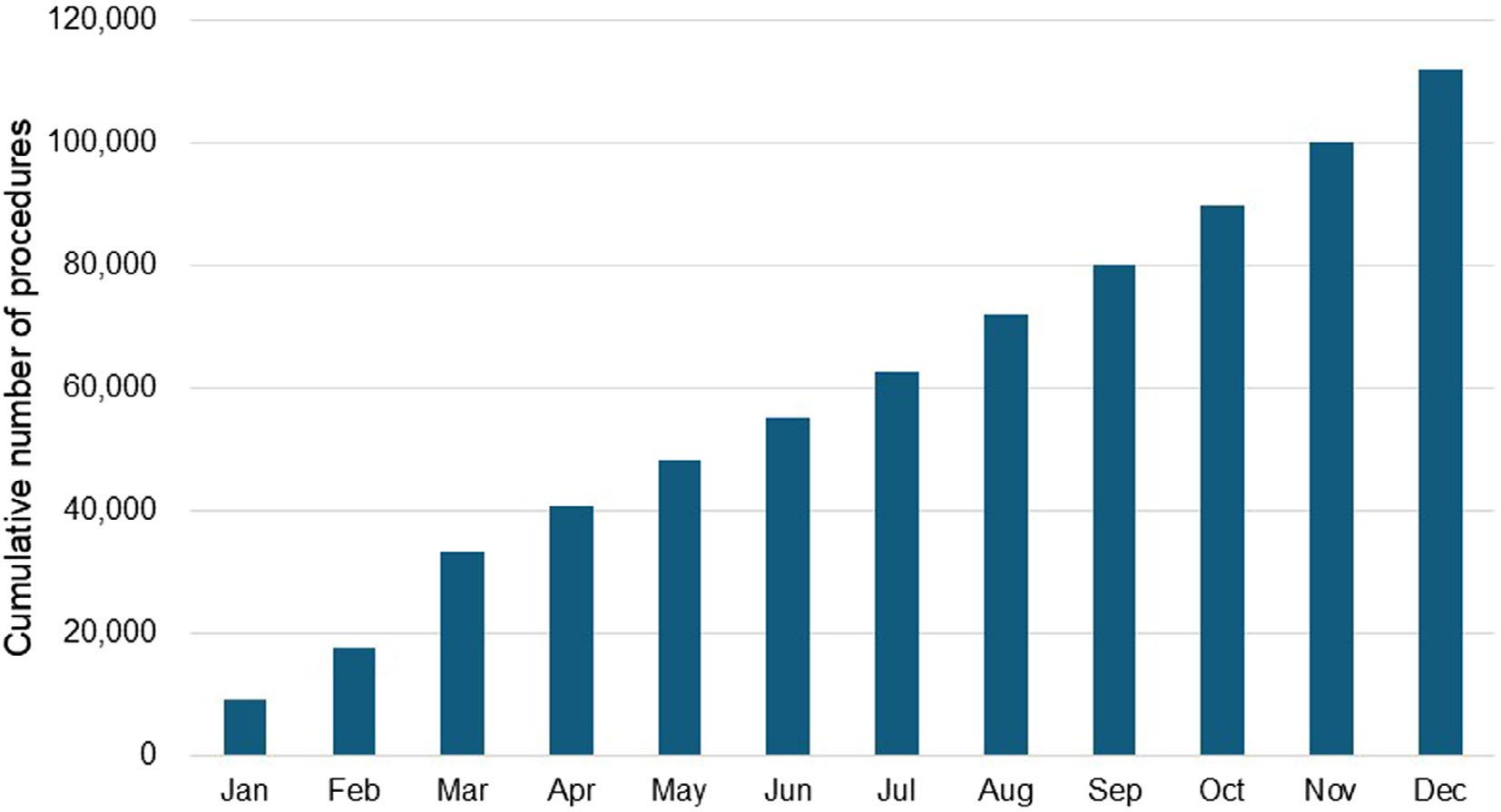
Cumulative number of registered procedures (blue bars) during the year of 2024.

**FIGURE 2:**
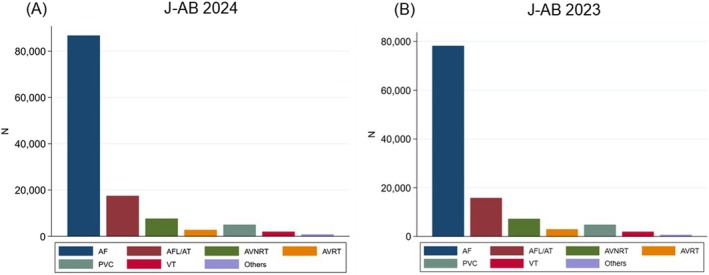
The number and rate of the target arrhythmias in the J‐AB 2024 (112 151 procedures); (A) and 2023 (102 584 procedures); (B). AF, atrial fibrillation; AFL, atrial flutter; AT, atrial tachycardia; AVNRT, atrioventricular nodal reentrant tachycardia; AVRT, atrioventricular reentrant tachycardia; PVC, premature ventricular contraction; VT, ventricular tachycardia.

**TABLE 1:** Patient characteristics.

	All procedures	Atrial Fibrillation (AF)			Atrial flutter (AFL)/Atrial Tachycardia (AT)				Atrioventricular nodal reentrant tachycardia	Atrioventricular reentrant tachycardia	Premature ventricular contraction	Ventricular tachycardia (VT)		
		All AF	Paroxysmal AF (PAF)	Non‐PAF	All AFL/AT	IVC‐TV isthmus dependent AFL	Uncommon AFL macro AT	Focal AT				Idiopathic VT	VT due to ischemic cardiomyopathy	VT due to nonischemic cardiomyopathy
*N*	1,12 151	86 779	47 767	39 012	17 498	11 205	4621	3260	7665	2801	5058	724	570	575
Age, mean ± SD	67.5 ± 12.9	69.1 ± 10.6	69.0 ± 11.0	69.3 ± 10.2	70.1 ± 12.3	70.1 ± 11.6	71.9 ± 11.8	67.9 ± 14.9	60.0 ± 16.6	47.5 ± 20.5	59.3 ± 15.9	56.2 ± 19.4	69.4 ± 10.5	64.8 ± 12.7
Gender, male	73 155 (65.2)	58 520 (67.4)	30 214 (63.3)	28 306 (72.6)	11 843 (67.7)	8457 (75.5)	2652 (57.4)	1582 (48.5)	3244 (42.3)	1768 (63.1)	2949 (58.3)	493 (68.1)	527 (92.5)	470 (81.7)
Heart diseases														
IHD														
No	100 880 (90.0)	78 150 (90.1)	42 954 (89.9)	35 196 (90.2)	15 478 (88.5)	9860 (88.0)	4028 (87.2)	3006 (92.2)	7285 (95.0)	2707 (96.6)	4568 (90.3)	655 (90.5)	—	528 (91.8)
Yes	9862 (8.8)	7562 (8.7)	4216 (8.8)	3346 (8.6)	1848 (10.6)	1236 (11.0)	544 (11.8)	236 (7.2)	268 (3.5)	61 (2.2)	450 (8.9)	66 (9.1)	—	35 (6.1)
Unknown	1409 (1.3)	1067 (1.2)	597 (1.2)	470 (1.2)	172 (1.0)	109 (1.0)	49 (1.1)	18 (0.6)	112 (1.5)	33 (1.2)	40 (0.8)	3 (0.4)	—	12 (2.1)
Cardiomyopathy														
No	102 958 (91.8)	79 778 (91.9)	45 190 (94.6)	34 588 (88.7)	15 722 (89.9)	10 073 (89.9)	4021 (87.0)	3016 (92.5)	7498 (97.8)	2733 (97.6)	4615 (91.2)	646 (89.2)	534 (93.7)	—
Yes	7887 (7.0)	5986 (6.9)	2021 (4.2)	3965 (10.2)	1619 (9.3)	1038 (9.3)	548 (11.9)	228 (7.0)	99 (1.3)	40 (1.4)	406 (8.0)	70 (9.7)	25 (4.4)	—
Unknown	1306 (1.2)	1015 (1.2)	556 (1.2)	459 (1.2)	157 (0.9)	94 (0.8)	52 (1.1)	16 (0.5)	68 (0.9)	28 (1.0)	37 (0.7)	8 (1.1)	11 (1.9)	—
Valve disease														
No	103 010 (91.8)	79 659 (91.8)	44 736 (93.7)	34 923 (89.5)	15 292 (87.4)	9927 (88.6)	3678 (79.6)	2937 (90.1)	7426 (96.9)	2734 (97.6)	4846 (95.8)	685 (94.6)	497 (87.2)	479 (83.3)
Yes	8003 (7.1)	6227 (7.2)	2499 (5.2)	3728 (9.6)	2085 (11.9)	1208 (10.8)	900 (19.5)	313 (9.6)	170 (2.2)	38 (1.4)	185 (3.7)	37 (5.1)	65 (11.4)	87 (15.1)
Unknown	1138 (1.0)	893 (1.0)	532 (1.1)	361 (0.9)	121 (0.7)	70 (0.6)	43 (0.9)	10 (0.3)	69 (0.9)	29 (1.0)	27 (0.5)	2 (0.3)	8 (1.4)	9 (1.6)
CHD														
No	109 567 (97.7)	85 089 (98.1)	46 830 (98.0)	38 259 (98.1)	16 712 (95.5)	10 743 (95.9)	4250 (92.0)	3147 (96.5)	7542 (98.4)	2734 (97.6)	4987 (98.6)	712 (98.3)	559 (98.1)	559 (97.2)
Yes	1464 (1.3)	797 (0.9)	410 (0.9)	387 (1.0)	654 (3.7)	398 (3.6)	312 (6.8)	102 (3.1)	65 (0.8)	41 (1.5)	46 (0.9)	9 (1.2)	2 (0.4)	7 (1.2)
Unknown	1120 (1.0)	893 (1.0)	527 (1.1)	366 (0.9)	132 (0.8)	64 (0.6)	59 (1.3)	11 (0.3)	58 (0.8)	26 (0.9)	25 (0.5)	3 (0.4)	9 (1.6)	9 (1.6)

Abbreviations: CHD, congenital heart disease; IHD, ischemic heart disease; SD, standard deviation.

**TABLE 2:** Acute outcomes.

2024	*n* (%)	2023	*n* (%)	2024%–2023% change
Pulmonary vein isolation of atrial fibrillation	*n* = 86 633	Pulmonary vein isolation of atrial fibrillation	*n* = 77 906	
**Ablation System**		**Ablation System**		
RF	65 810 (76.0%)	RF alone	62 223 (79.9)	−3.9
Ballon ablation (Cryo, hot, laser)	22 641 (26.1%)	Ballon ablation (Cryo, hot, laser)	21 629 (27.8)	−1.7
Pulse field ablation	4498 (5.2%)	Pulse field ablation	0 (0.0)	+5.2
**Patient with a first session (*n* = 55 170)**	*N* = 69 825	**Patient with a first session**	*n* = 62 856	
Success	69 402 (99.4%)	Success	62 499 (99.4%)	0.0
Unsuccess	357 (0.5%)	Unsuccess	357 (0.6%)	−0.1
Unknown or others	66 (0.1%)	Unknown	0 (0.0%)	+0.1
**Patient with second session**	*N* = 13 419	**Patient with second session**	*n* = 12 106	
Success	9255 (69.0%)	Success	8478 (70.0%)	−1.0
Unsuccess	36 (0.3%)	Unsuccess	29 (0.2%)	+0.1
Already isolated	4128 (30.8%)	Already isolated	3599 (29.7%)	+1.1
**Patient with ≥third session**	*N* = 3360	**Patient with≥third session**	*n* = 2927	
Success	1354 (40.3%)	Success	1120 (38.3%)	+2.0
Unsuccess	9 (0.3%)	Unsuccess	9 (0.3%)	0.0
Already isolated	1997 (59.4%)	Already isolated	1798 (61.4%)	−2.0
**IVC‐TV isthmus dependent atrial flutter**	*N* = 11 205	**IV‐TV isthmus dependent atrial flutter (*n* = 9605)**	*n* = 10 086	
Success	11 147 (99.5%)	Success	10 037 (99.5%)	0.0
Unsuccess	58 (0.5%)	Unsuccess	49 (0.5%)	0.0
**Uncommon atrial flutter/atrial tachycardia**	*N* = 4621	**Uncommon atrial flutter/atrial tachycardia (*n* = 3957)**	*n* = 4039	
Complete success	4076 (88.2%)	Complete success	3517 (87.1%)	+1.1
Partial success	403 (8.7%)	Partial success	382 (9.5%)	−0.8
Unsuccess	113 (2.4%)	Unsuccess	103 (2.6%)	−0.2
Unknown or others	29 (0.6%)	Unknown	37 (0.9%)	−0.3
**Focal atrial tachycardia**	*N* = 3260	**Focal atrial tachycardia (*n* = 2894)**	*n* = 3054	
Complete success	2827 (86.7%)	Complete success	2601 (85.2%)	+1.5
Partial success	301 (9.2%)	Partial success	322 (10.5%)	−1.3
Unsuccess	88 (2.7%)	Unsuccess	89 (2.9%)	−0.2
Unknown or others	44 (1.3%)	Unknown	42 (1.4%)	−0.1
**Atrioventricular nodal reentrant tachycardia by slow‐fast**	*N* = 6544	**Atrioventricular nodal reentrant tachycardia by slow‐fast (*n* = 5534)**	*n* = 6248	
Complete success	6380 (97.5%)	Complete success	6108 (97.8%)	−0.3
Partial success	123 (1.9%)	Partial success	108 (1.7%)	+0.2
Unsuccess	23 (0.4%)	Unsuccess	16 (0.3%)	+0.1
Unknown or others	18 (0.3%)	Unknown	16 (0.3%)	0.0
**Atrioventricular nodal reentrant tachycardia by fast‐slow**	*N* = 743	**Atrioventricular nodal reentrant tachycardia by fast‐slow (*n* = 573)**	*n* = 702	
Complete success	709 (95.4%)	Complete success	674 (96.0%)	−0.6
Partial success	25 (3.4%)	Partial success	19 (2.7%)	+0.7
Unsuccess	5 (0.7%)	Unsuccess	3 (0.4%)	+0.3
Unknown or others	4 (0.5%)	Unknown	6 (0.9%)	−0.4
**Atrioventricular nodal reentrant tachycardia by slow‐slow**	*N* = 544	**Atrioventricular nodal reentrant tachycardia by slow‐slow (*n* = 356)**	*n* = 476	
Complete success	525 (96.5%)	Complete success	448 (94.1%)	+2.4
Partial success	14 (2.6%)	Partial success	18 (3.8%)	−1.2
Unsuccess	4 (0.7%)	Unsuccess	5 (1.1%)	−0.4
Unknown or others	1 (0.2%)	Unknown	5 (1.1%)	−0.9
**Atrioventricular reentrant tachycardia by kent**	*N* = 2801	**Atrioventricular reentrant tachycardia by kent (*n* = 2670)**	*n* = 2981	
Complete success	2681 (97.3%)	Complete success	2819 (96.5%)	+0.8
Unsuccess	50 (1.8%)	Unsuccess	71 (2.4%)	−0.6
Unknown or others	25 (0.9%)	Unknown	31 (1.1%)	−0.2
**Premature ventricular contraction**	*N* = 5058	**Premature ventricular contraction (*n* = 4314)**	*n* = 4849	
Complete success	3915 (77.4%)	Complete success	3763 (77.6%)	−0.2
Partial success	890 (17.6%)	Partial success	837 (17.3%)	+0.3
Unsuccess	211 (4.2%)	Unsuccess	198 (4.1%)	+0.1
Unknown or others	42 (0.8%)	Unknown	51 (1.1%)	−0.3
**Idiopathic ventricular tachycardia**	*N* = 724	**Idiopathic ventricular tachycardia (*n* = 778)**	*n* = 792	
Complete success	585 (80.8%)	Complete success	643 (81.2%)	−0.4
Partial success	96 (13.3%)	Partial success	99 (12.5%)	+0.8
Unsuccess	23 (3.2%)	Unsuccess	32 (4.0%)	−0.8
Unknown or others	20 (2.8%)	Unknown	18 (2.3%)	+0.5
**Ventricular tachycardia due to ischemic cardiomyopathy**	*N* = 570	**Ventricular tachycardia due to ischemic cardiomyopathy (*n* = 459)**	*n* = 525	
Complete success	423 (74.2%)	Complete success	384 (73.1%)	+1.1
Partial success	119 (20.9%)	Partial success	110 (21.0%)	−0.1
Unsuccess	16 (2.8%)	Unsuccess	16 (3.0%)	−0.2
Unknown or others	12 (2.1%)	Unknown	15 (2.9%)	−0.8
**Ventricular tachycardia due to nonischemic cariomyopathy**	*N* = 575	**Ventricular tachycardia due to nonischemic cariomyopathy (*n* = 570)**	*n* = 523	
Complete success	381 (66.3%)	Complete success	312 (59.7%)	+6.6
Partial success	149 (25.9%)	Partial success	165 (31.5%)	−5.6
Unsuccess	26 (4.5%)	Unsuccess	30 (5.7%)	−1.2
Unknown or others	19 (3.3%)	Unknown	16 (3.1%)	+0.2

Abbreviations: IVC, inferior vena cava; RF, radiofrequency ablation; TV, tricuspid valve.

**TABLE 3:** Acute complications.

	2024		2023		2024%–2023% change	
	All patient	AF	All patient	AF	All patient	AF
*N*	1,12 151	86 779	1,02584	78 196		
Complications during hospitalization	2255 (2.01%)	1834 (2.11%)	2101 (2.05%)	1688 (2.16%)	−0.04	−0.05
Major bleeding (BARC≧ 2)	831 (0.74%)	638 (0.74%)	784 (0.76%)	576 (0.74%)	−0.02	0.00
Cardiac tamponade	495 (0.44%)	350 (0.40%)	483 (0.47%)	335 (0.43%)	−0.03	−0.03
Embolism	140 (0.12%)	122 (0.14%)	129 (0.13%)	106 (0.14%)	−0.01	0.00
Phrenic nerve paralysis	328 (0.29%)	320 (0.37%)	326 (0.32%)	320 (0.41%)	−0.03	−0.04
Esophagus	92 (0.08%)	91 (0.10%)	110 (0.11%)	110 (0.14%)	−0.03	−0.04
Gastric hypomotility	82 (0.07%)	82 (0.09%)	93 (0.09%)	93 (0.12%)	−0.02	−0.03
Pericardities	92 (0.08%)	81 (0.09%)	78 (0.08%)	64 (0.08%)	0.00	+0.01
Sick sinus syndrome	105 (0.09%)	87 (0.10%)	138 (0.13%)	113 (0.14%)	−0.04	−0.04
Atrioventricular block	76 (0.07%)	21 (0.02%)	66 (0.06%)	22 (0.03%)	+0.01	−0.01
Death during hospitalization	133 (0.12%)	61 (0.07%)	121 (0.12%)	50 (0.06%)	0.00	+0.01
Cardiac death	71 (0.06%)	27 (0.03%)	66 (0.06%)	19 (0.02%)	0.00	+0.01
Related to ablation therapy	2 (0.002%)	2 (0.002%)	7 (0.007%)	3 (0.004%)	−0.01	0.00
Non cardiac death	62 (0.06%)	34 (0.04%)	55 (0.05%)	31 (0.04%)	+0.01	0.00
Related to ablation therapy	0 (0.000%)	0 (0.000%)	3 (0.003%)	3 (0.004%)	0.00	0.00

## Funding

This work was supported by the Japanese Heart Rhythm Society.

## Ethics Statement

This study was approved by the central ethics review board of the Japanese Heart Rhythm Society (no. 2021001, approved at Dec 16, 2021).

## Conflicts of Interest

Kengo Kusano: Speaker honoraria from DAIICHI SANKYO COMPANY Ltd., Medtronic Japan, and research grants from Medtronic Japan, GE Precision Healthcare LLC, JSR, Blue Industries, Q'sfix, Biotronik Japan, and Boston Scientific Japan. Koichi Inoue: Speaker honoraria from DAIICHI SANKYO COMPANY Ltd., Johnson & Johnson K.K., Medtronic Japan, and Boston Scientific Japan. and research grant from Johnson & Johnson K.K., and Japan Lifeline. Koshiro Kanaoka: Speaker honoraria from Eli Lilly Japan and Otsuka Pharmaceutical Co. and consultation fee from Johnson & Johnson K.K. Koji Miyamoto received funding/grants from Medtronic Japan, Biosense Webster, Abbott Medical Japan LLC, and Boston Scientific Japan; honoraria/speakers' bureaus from Medtronic Japan, Biosense Webster, Abbott Medical Japan LLC, and Boston Scientific Japan; and consultancies from Medtronic Japan, Abbott Medical Japan LLC, and Boston Scientific Japan outside the submitted work and is affiliated with a department endowed by Medtronic Japan outside the submitted work. Yasuo Okumura has received research funding from Medtronic Japan Co. Ltd., MicroPort CRM Japan, and Bayer Healthcare; and has accepted remuneration from AstraZeneca K.K. and Johnson & Johnson K.K. He is affiliated with endowed departments supported by Abbott Medical Japan LLC, Boston Scientific Japan K.K., Medtronic Japan Co. Ltd., Japan Lifeline Co. Ltd., and Biotronik Japan. Kazuhiro Satomi received research funding irrelevant to this study from Abbott Medical Japan LLC, Boston Scientific Japan, Biotronik Japan, and lecture fees from Medtronic Japan and Japan Lifeline. Seiji Takatsuki belongs to Advanced Cardiac Arrhythmia Therapeutics Endowed Research Course, which is supported by Medtronic Japan, Japan Lifeline, Boston Scientific Japan, Abbot Japan and Biotronik Japan. He is received research fundings irrelevant to this study from Nippon Boehringer Ingelheim, Japan Lifeline, Boston Scientific Japan, and lecture fees from Medtronic Japan, Japan Lifeline, DAIICHI SANKYO COMPANY Ltd., Boston Scientific Japan, Bayer Yakuhin, Biotronik Japan, Abbot Japan, and Nihon‐koden. Masaharu Masuda received research funding irrelevant to this study from Johnson & Johnson K.K. and lecture fees from Medtronic Japan, DAIICHI SANKYO COMPANY, and Boston Scientific Japan. Teiichi Yamane: Speaker honoraria from Medtronic Japan, Johnson and Johnson K.K., and BEG company and research grants from Japan Lifeline. Wataru Shimizu has received speaker honoraria from DAIICHI SANKYO COMPANY Ltd.; Johnson & Johnson K.K.; Asahi Kasei ZOLL Medical Corporation; Boston Scientific Japan K.K.; Japan Lifeline Co. Ltd.; and Medtronic Japan Co. Ltd. Dr. Hiroshi Tada is affiliated with endowed departments sponsored by BIOTRONIK Japan Inc., DVx Inc., ALVAUS Co. Ltd., and Fukuda Denshi Co. Ltd. Dr. Hiroshi Tada received honoraria for lectures or speakers bureaus from DAIICHI SANKYO COMPANY Ltd.; Medtronic Japan Co. Ltd.; BIOTRONIK Japan Inc.; Boston Scientific Japan K.K. Dr. Tada also received grants (Investigator‐initiated study unrelated to the manuscript topic) from Abbott Medical Japan LLC. None: K.K., Y.I., K.N., Y.I., S.C.K.

## Data Availability

The data that support the findings of this study are available on request from the corresponding author. The data are not publicly available due to privacy or ethical restrictions.
